# Supplementary material for: Pyruvate Dehydrogenase Kinase 1 inhibition mediated oxidative phosphorylation enhancement in cartilage promotes osteoarthritis progression
Source: BMC Musculoskelet Disord. 2023 Jul 20;24:597. doi: 10.1186/s12891-023-06585-6 (PMC10357736; doi:10.1186/s12891-023-06585-6)
Supplement: Supplementary file 1 — Additional file 1. [file 12891_2023_6585_MOESM1_ESM.zip › KEGG.Gsea.1653622432464/KEGG_DORSO_VENTRAL_AXIS_FORMATION.html]

Details for gene set KEGG\_DORSO\_VENTRAL\_AXIS\_FORMATION[GSEA]

|  || Dataset | 55457\_collapsed\_to\_symbols.cls.cls #OA\_versus\_normal.cls.cls #OA\_versus\_normal\_repos |
| Phenotype | cls.cls#OA\_versus\_normal\_repos |
| Upregulated in class | normal |
| GeneSet | KEGG\_DORSO\_VENTRAL\_AXIS\_FORMATION |
| Enrichment Score (ES) | -0.6147877 |
| Normalized Enrichment Score (NES) | -1.8948643 |
| Nominal p-value | 0.003539823 |
| FDR q-value | 0.014061615 |
| FWER p-Value | 0.045 |
Table: GSEA Results Summary

  

Fig 1: Enrichment plot: KEGG\_DORSO\_VENTRAL\_AXIS\_FORMATION      
 Profile of the Running ES Score & Positions of GeneSet Members on the Rank Ordered List

  

| SYMBOL | TITLE | RANK IN GENE LIST | RANK METRIC SCORE | RUNNING ES | CORE ENRICHMENT || 1 | MAPK3 | mitogen-activated protein kinase 3 [Source:HGNC Symbol;Acc:HGNC:6877] | 757 | 0.579 | 0.0233 | No |
| 2 | GRB2 | growth factor receptor bound protein 2 [Source:HGNC Symbol;Acc:HGNC:4566] | 5697 | 0.016 | -0.3578 | No |
| 3 | PIWIL1 | piwi like RNA-mediated gene silencing 1 [Source:HGNC Symbol;Acc:HGNC:9007] | 6144 | -0.016 | -0.3902 | No |
| 4 | CPEB1 | cytoplasmic polyadenylation element binding protein 1 [Source:HGNC Symbol;Acc:HGNC:21744] | 6435 | -0.038 | -0.4074 | No |
| 5 | MAPK1 | mitogen-activated protein kinase 1 [Source:HGNC Symbol;Acc:HGNC:6871] | 6853 | -0.068 | -0.4302 | No |
| 6 | NOTCH3 | notch receptor 3 [Source:HGNC Symbol;Acc:HGNC:7883] | 7894 | -0.147 | -0.4900 | No |
| 7 | SOS2 | SOS Ras/Rho guanine nucleotide exchange factor 2 [Source:HGNC Symbol;Acc:HGNC:11188] | 8058 | -0.160 | -0.4800 | No |
| 8 | ETS1 | "ETS proto-oncogene 1, transcription factor [Source:HGNC Symbol;Acc:HGNC:3488]" | 9795 | -0.301 | -0.5721 | Yes |
| 9 | MAP2K1 | mitogen-activated protein kinase kinase 1 [Source:HGNC Symbol;Acc:HGNC:6840] | 10197 | -0.340 | -0.5550 | Yes |
| 10 | KRAS | "KRAS proto-oncogene, GTPase [Source:HGNC Symbol;Acc:HGNC:6407]" | 10544 | -0.377 | -0.5283 | Yes |
| 11 | NOTCH2 | notch receptor 2 [Source:HGNC Symbol;Acc:HGNC:7882] | 10720 | -0.395 | -0.4858 | Yes |
| 12 | ETS2 | "ETS proto-oncogene 2, transcription factor [Source:HGNC Symbol;Acc:HGNC:3489]" | 11057 | -0.439 | -0.4497 | Yes |
| 13 | PIWIL2 | piwi like RNA-mediated gene silencing 2 [Source:HGNC Symbol;Acc:HGNC:17644] | 11219 | -0.460 | -0.3970 | Yes |
| 14 | NOTCH4 | notch receptor 4 [Source:HGNC Symbol;Acc:HGNC:7884] | 11441 | -0.495 | -0.3440 | Yes |
| 15 | ETV7 | ETS variant transcription factor 7 [Source:HGNC Symbol;Acc:HGNC:18160] | 11872 | -0.566 | -0.2972 | Yes |
| 16 | SOS1 | SOS Ras/Rac guanine nucleotide exchange factor 1 [Source:HGNC Symbol;Acc:HGNC:11187] | 12041 | -0.603 | -0.2246 | Yes |
| 17 | EGFR | epidermal growth factor receptor [Source:HGNC Symbol;Acc:HGNC:3236] | 12109 | -0.622 | -0.1416 | Yes |
| 18 | ETV6 | ETS variant transcription factor 6 [Source:HGNC Symbol;Acc:HGNC:3495] | 12231 | -0.654 | -0.0582 | Yes |
| 19 | NOTCH1 | notch receptor 1 [Source:HGNC Symbol;Acc:HGNC:7881] | 12555 | -0.776 | 0.0268 | Yes |
Table: GSEA details [plain text format]

  

Fig 2: KEGG\_DORSO\_VENTRAL\_AXIS\_FORMATION      
 Blue-Pink O' Gram in the Space of the Analyzed GeneSet

  

Fig 3: KEGG\_DORSO\_VENTRAL\_AXIS\_FORMATION: Random ES distribution      
 Gene set null distribution of ES for **KEGG\_DORSO\_VENTRAL\_AXIS\_FORMATION**
